# Supplementary material for: Learned adaptive multiphoton illumination microscopy for large-scale immune response imaging
Source: Nat Commun. 2021 Mar 26;12:1916. doi: 10.1038/s41467-021-22246-5 (PMC7997974; doi:10.1038/s41467-021-22246-5)
Supplement: Supplementary file 1 — Supplementary Information [file 41467_2021_22246_MOESM1_ESM.pdf]

Supplementary materials for: Learned adaptive multiphoton illumination microscopy for large-scale immune response imaging

This PDF includes:

Supplementary Figures S1 to S10

List of supplementary figures:

S1: Nonuniform excitation across field on curved tissues

S2: Spatial light modulator test patterns

S3: Circuit diagram of time-realized spatial light modulator

S4: Spherical tissue ray-optics scattering model

S5: Curved samples require sub-exponential power increases with depth

S6: A comparison of different adaptive excitation strategies

S7: Registration as optimization

S8: Motion artifact correction and active learning-based cell detection

S9: Engineered features for cell classification

S10: Reorganization of lymph nodes 24 hours after immunization

S11: Dendritic cell motility changes in different anatomical locations

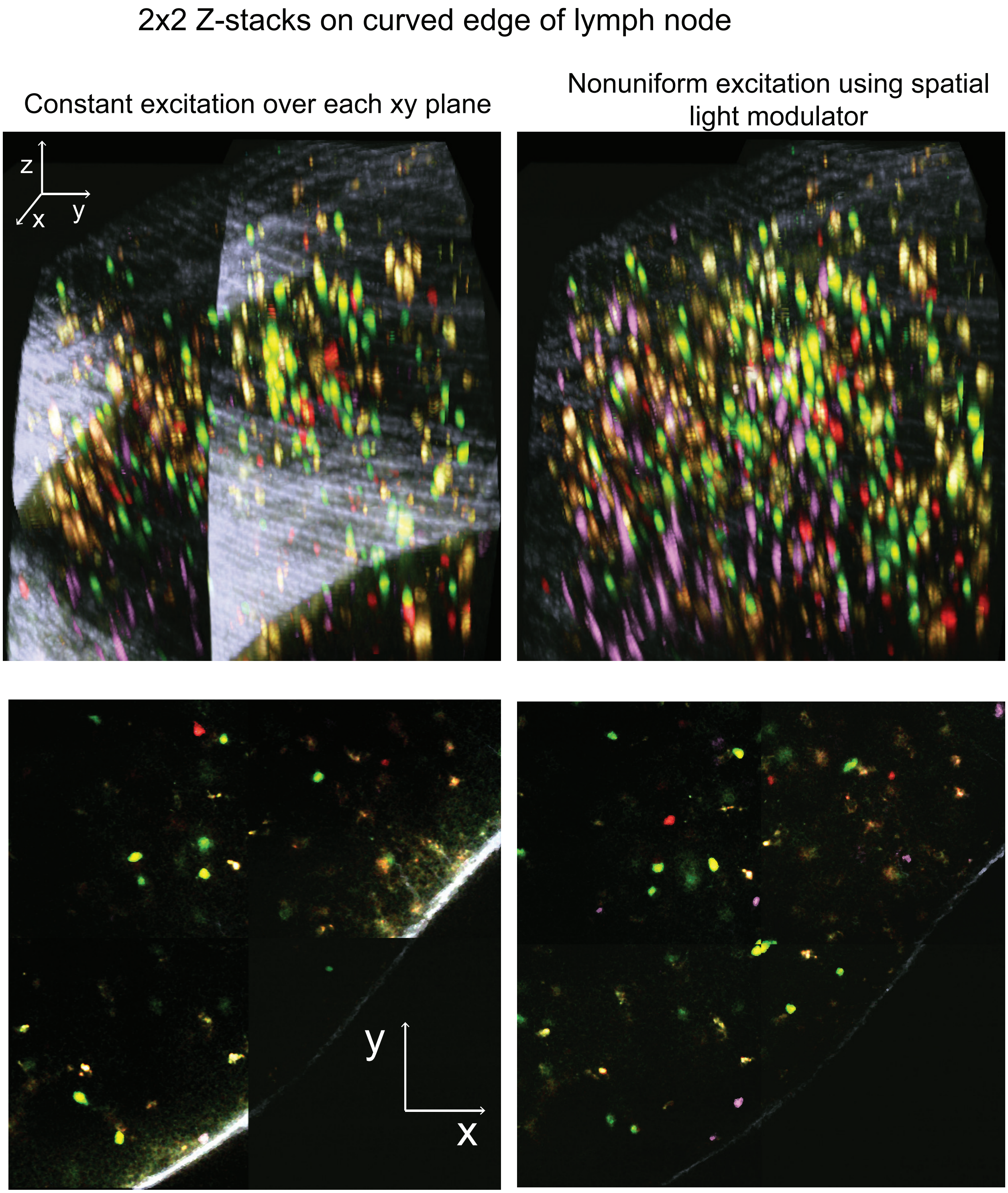

Supplementary Figure 1: Nonuniform excitation across field on curved tissue. Imaging into to curved tissue such as the edge of a lymph node requires variable excitation over the XY field of view. 3D view (top) and 2D slice (bottom) of a 2x2 grid of Z-stacks. Left, constant excitation power within each XY plane in each Z stack. Right, variable excitation power allows excitation to be set correctly for each point in XYZ field of view.

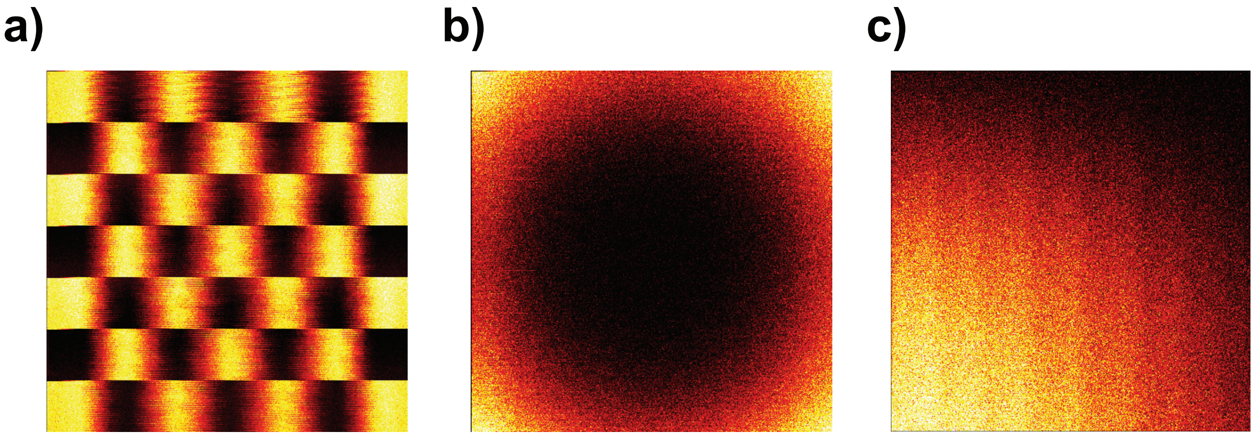

Supplementary Figure 2: Spatial Light Modulator Test Patterns. Images taken on flat fluorescent test slide with different patterns of excitation light. a) A checkerboard pattern demonstrating the difference in horizontal vs. vertical resolution. b) A vignetting compensation pattern, with more excitation at the edges of the field of view. c) A gradient across the field of view pattern.

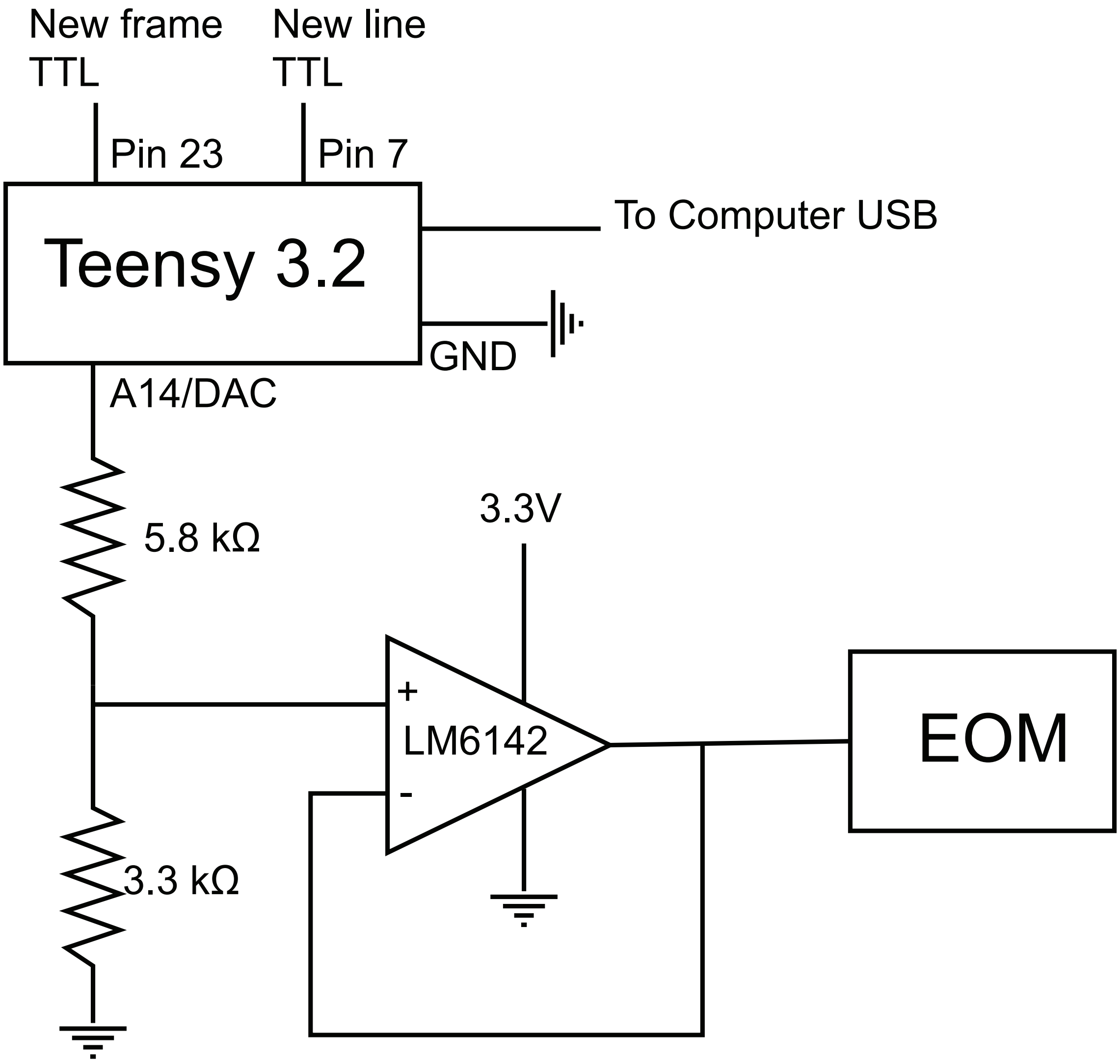

Supplementary Figure 3: Circuit diagram of time-realized spatial light modulator (TR-SLM). Wiring of circuit connecting Teensy 3.2 to electro-optic modulator (EOM) that controls excitation laser power via an op-amp. New frame TTL connects to a trigger that fires every time the raster scan pattern begins a new frame. New line TTL connects to a trigger that fires after resonant scanner completes a new line (which corresponds to two rows of pixels)

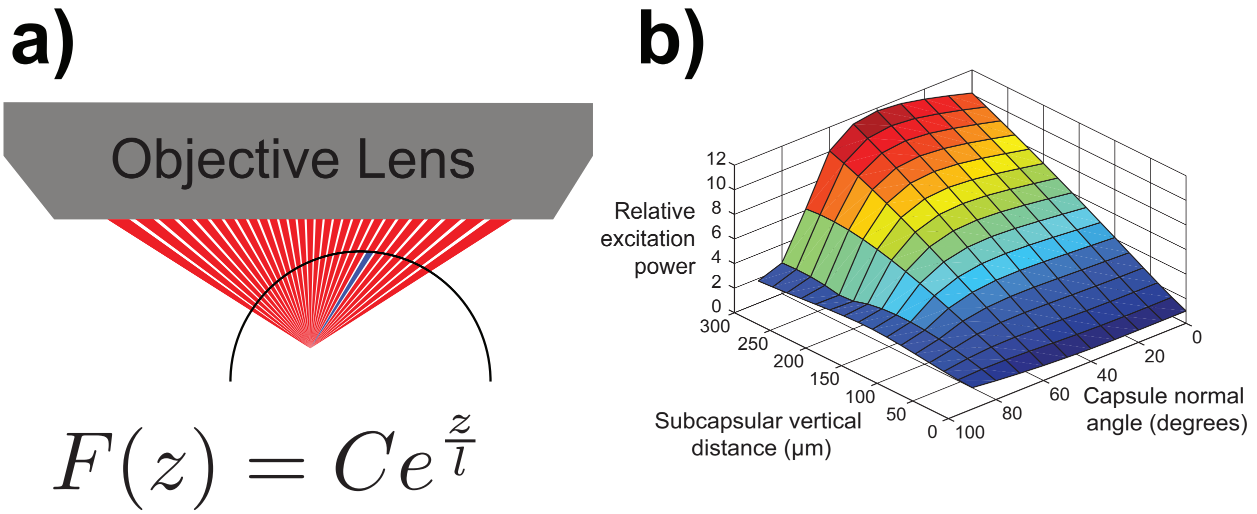

Supplementary Figure 4: Spherical tissue ray-optics scattering model. A previous scattering model used on the way to developing standard candle calibration. In this model, the tissue is assumed to be a sphere with homogeneous scattering potential. a) Fluorescence at the focal point is computed by integrating the contribution from every ray within the cone of the objective's numerical aperture. The contribution of each ray drops off with its propagation distance through tissue (z) as shown in the equation. b) The predictions of the model with parameters estimated for lymph node tissue. Relative excitation power is the inverse of the fraction of input power that makes it to the focal point. It is indexed by the vertical distance from the focal point to the top of the tissue, and the normal angle of the sphere directly above the focal point.

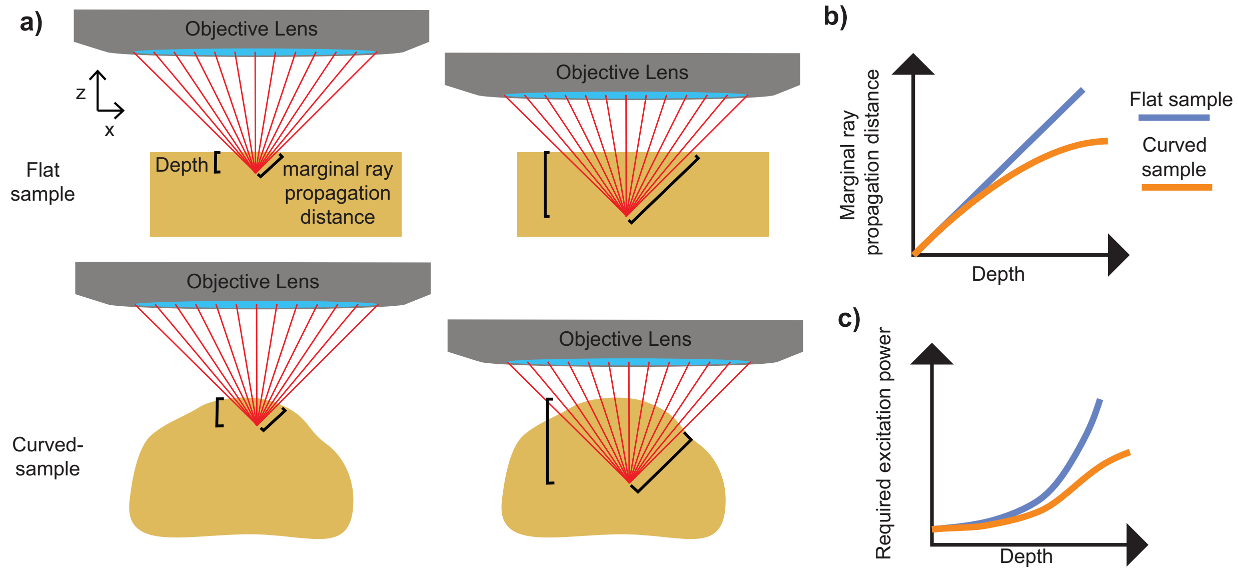

Supplementary Figure 5: Curved samples require sub-exponential power increases with depth. a, b) When focusing into a flat sample, the distance from the focal point to the top of the sample ("depth") and the distance travelled by the marginal ray through the sample increase linearly, in a curved sample, the distance travelled by the marginal ray increases sub-linearly. c) As a result, the curved sample requires sub-exponential increases in laser power to maintain signal with depth.

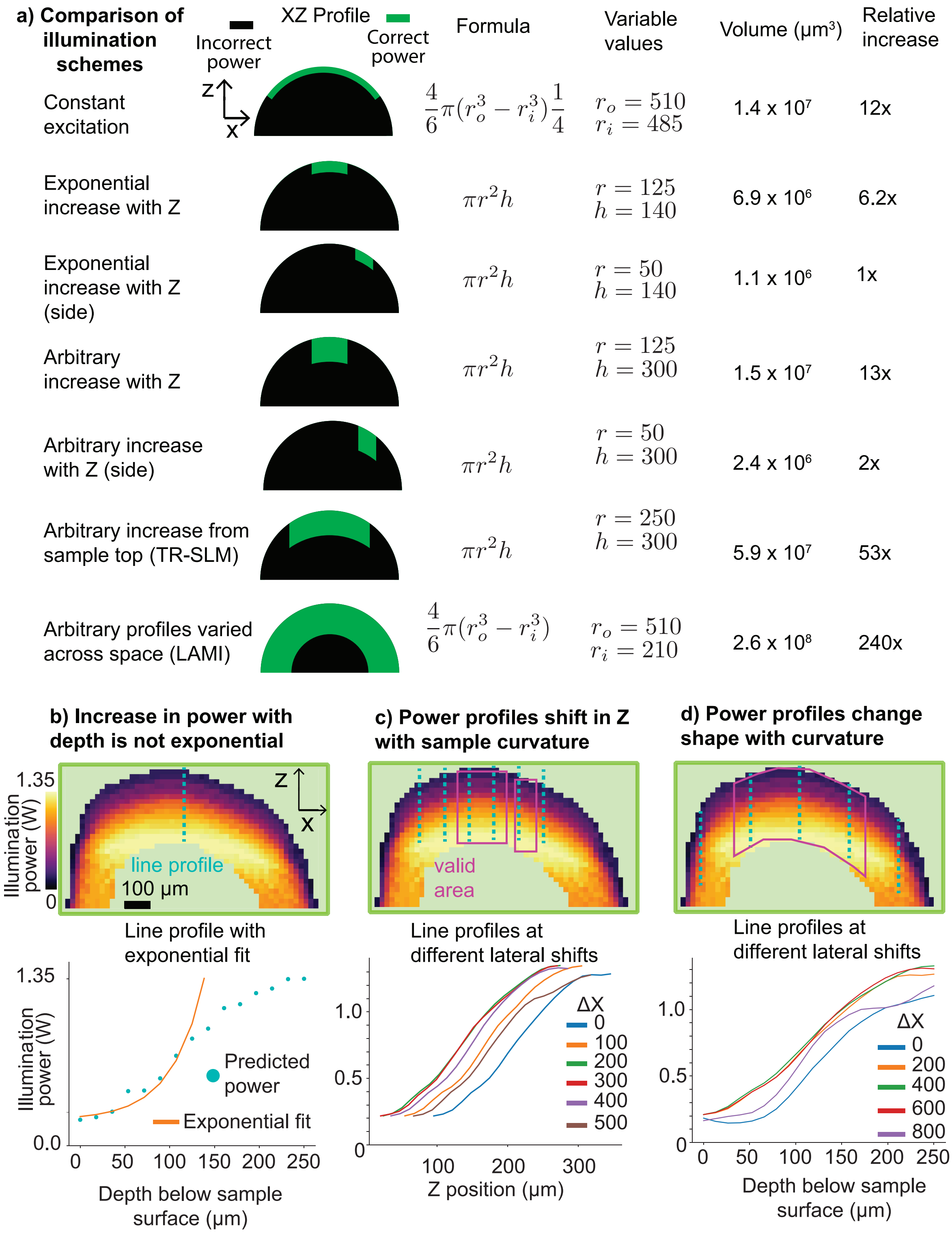

Supplementary Figure 6: A comparison of different adaptive excitation strategies. a) Overview of various adaptive excitation strategies, including details of calculations for the total volume each can image. The top 5 rows are strategies that are employed on existing multiphoton microscopes. The bottom two are enabled by the development of time-realized spatial light modulator (TR-SLM) and TR-SLM + learned adaptive multiphoton illumination (LAMI), respectively. Various values used in the calculations are derived from measurements shown in b-d. b) Top, XZ slice of excitation predicted by LAMI in a popliteal lymph node. Cyan dashed line shows profile, which is plotted with an exponential fit on the bottom. The excitation only follows an exponential profile for $\sim$140 μm. c) Excitation power Z profiles spaced at 100 μm intervals (cyan dashed lines and bottom plot). Magenta boxes show areas that can be imaged with an approximately constant profile in Z. d) Excitation power profiles starting from the top of the sample (cyan dashed lines and bottom plot). Moving towards the edges, the shape of the profiles noticeably changes. The magenta outlined region shows the region that can be imaged with a single excitation profile, applied starting at the top of the sample.

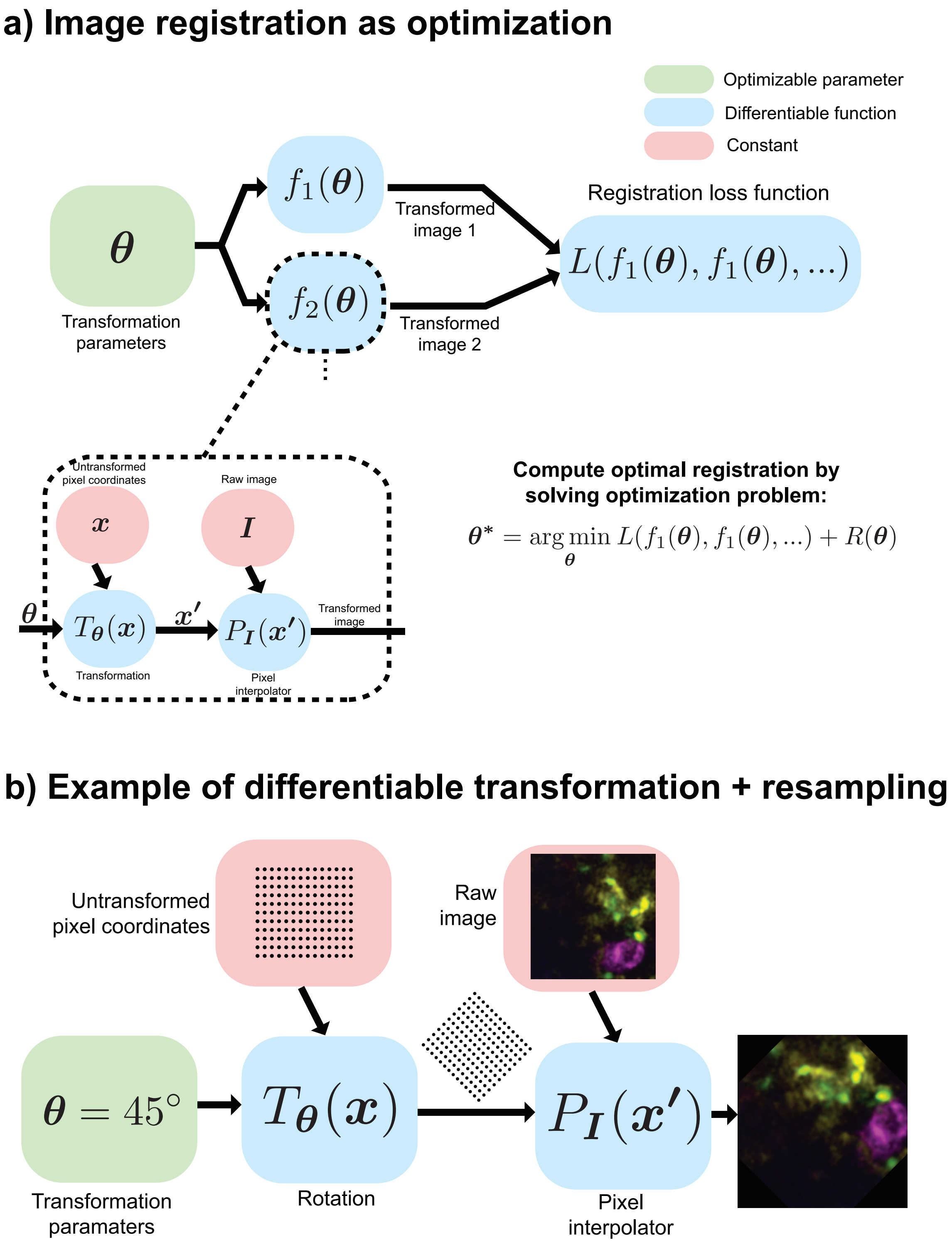

Supplementary Figure 7: Image registration formulated as iterative optimization
a) Overview of *maximum a posteriori* estimation for image registration. Differentiable transformations specific to each correction are used to resample raw image pixels, and fed into a loss function that quantified the quality of solution. b) Rotation as an example of a differentiable transformation.

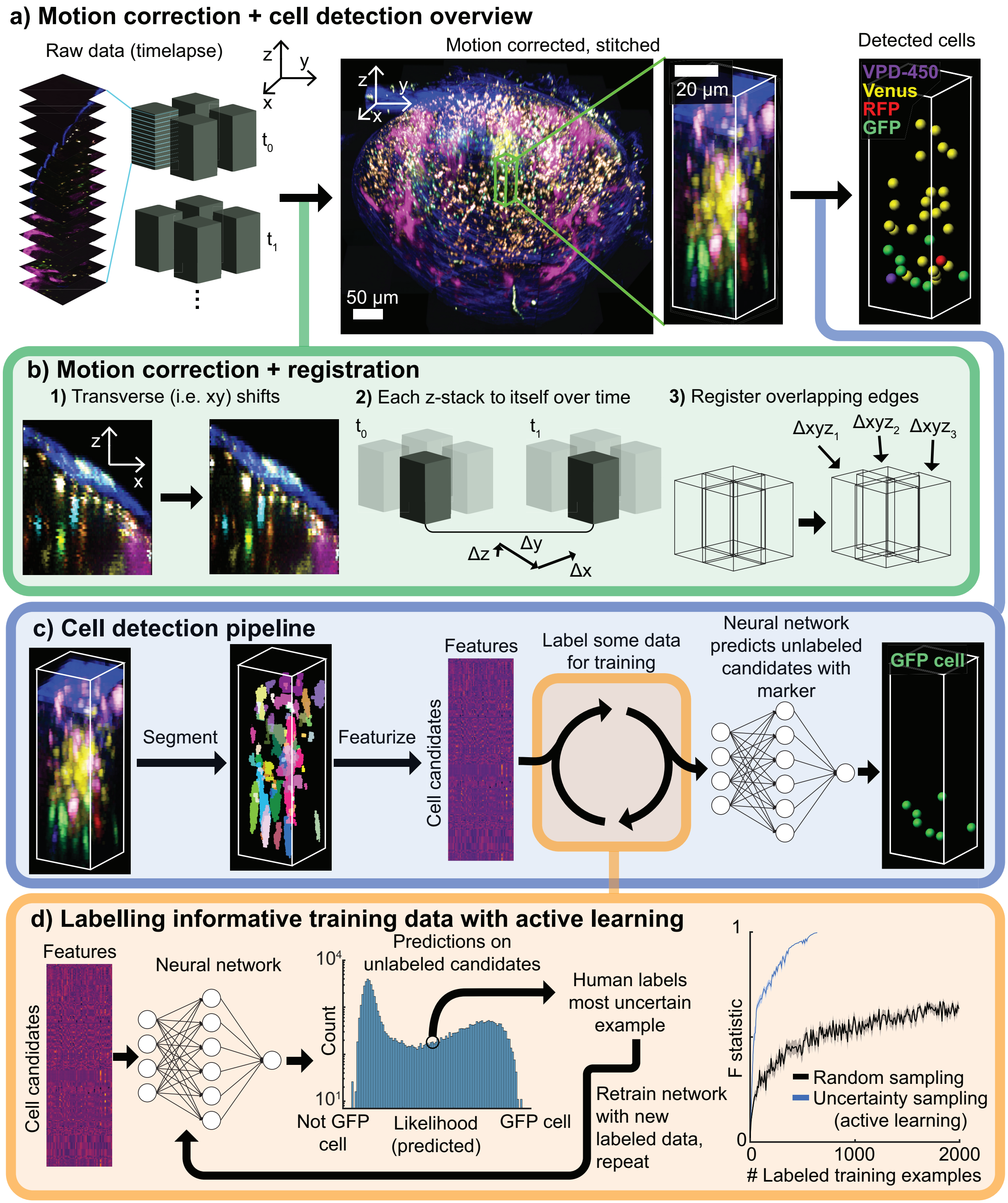

Supplementary Figure 8: Motion artifact correction and active learning-based cell detection a) Overview of data processing converting raw data of separate z-stacks into a single stitched and motion-corrected volume, followed detecting cells based on individual fluorescent protein expression. b) Motion correction and registration consisted of three types of corrections: 1) the XY movements within each slice were optimized. 2) Stacks at consecutive timepoints were registered to one another using cross correlation. 3) The alignment between stacks was optimized. c) Cell identification began by computing a 3D segmentation algorithm to identify candidate cells. Features were then computed for each candidate cell and fed into a classification neural network that predicts which candidates belong to the population of interest d) Active learning was used to label an informative training set. In this paradigm the classification network outputs a measure of certainty that each candidate is a cell or not. The most uncertain of these examples is selected for human labelling, the classification network is retrained, and the procedure is repeated. This enables selection of which candidates belong to population of interest (e.g. GFP). Right, active learning data labelling dramatically boosts classifier accuracy compared to randomly sampling and labelling data.

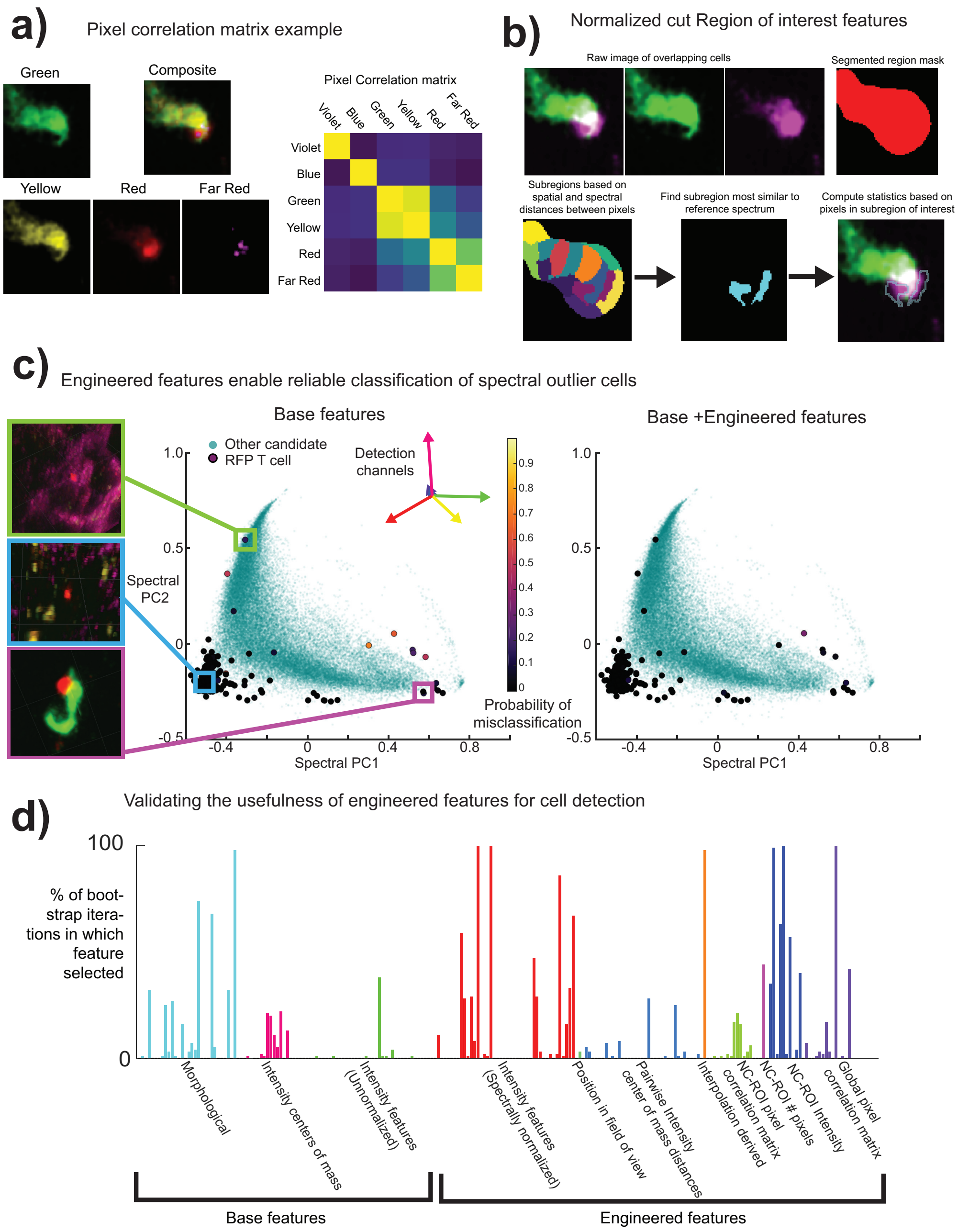

Supplementary Figure 9: Engineered features for cell classification a) The pairwise correlations between pixels of different channels. This provides a clear signal (i.e. distinct clusters in the correlation matrix) when a GFP and RFP labelled cell do not entirely spatially overlap. b) Normalized cut features: by breaking down an area of masked pixels (red, top right) into subregions (bottom left), a subregion that is most similar to a reference spectrum (i.e. the magenta cell) can be identified). c) Including engineered features enables robust identification of spectral outliers. Plots show all candidate cells plotted over first two principal components of the average color spectrum of RFP T cells. Left, spectral outliers (representative images shown on left) from the main cluster of T cells also tend to misclassified. Right, adding in engineered features to classification vastly improves the misclassification probability of these spectral outliers. d) Elastic net bootstrap analysis colored by feature class. Many classes of bootstrapped features were selected a high proportion of the time, validating their usefulness in this classification problem.

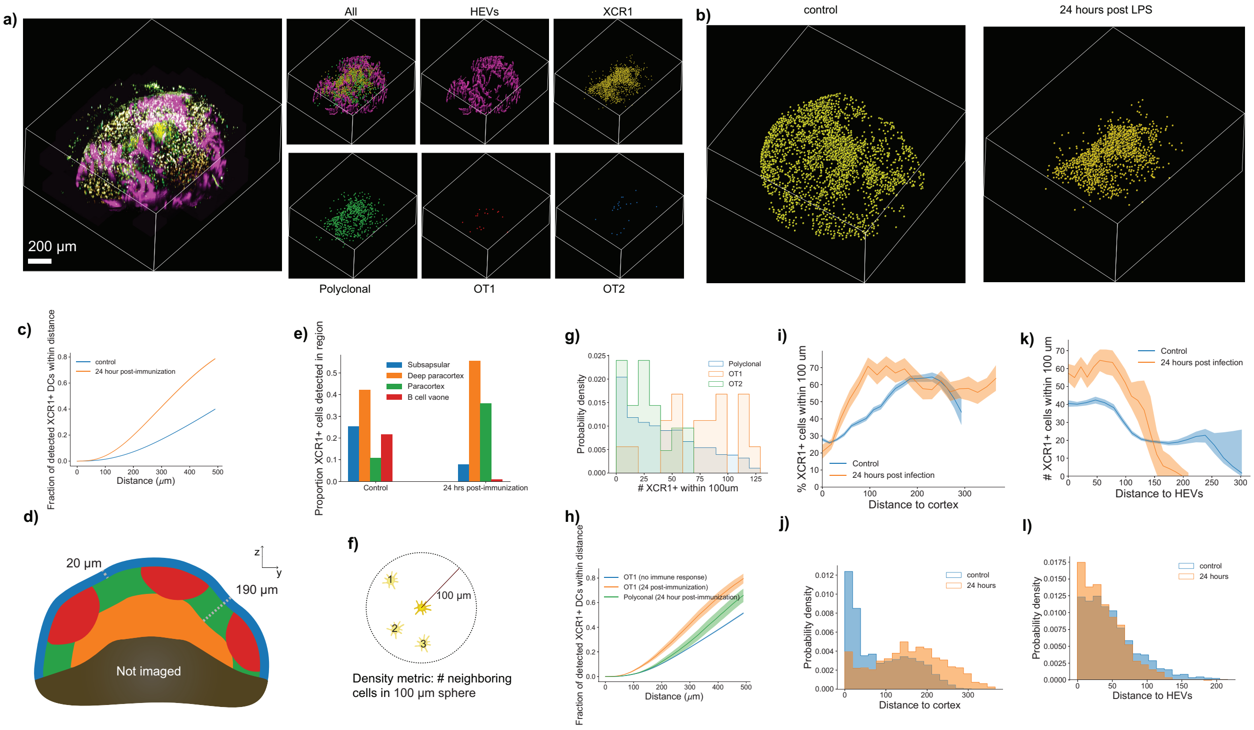

Supplementary Figure 10: Reorganization of cell population in the lymph node 24 hours after immunization a) Image data (left) and localizations of XCR1, Polyclonal, OT1, and OT2 as well as 3D segmentation of high endothelial venules. b) Localization of XCR1 cells in control condition and 24 hours after immunization. c) Amount of clustering as assessed by the mean fraction of XCR1 cells within different distances of XCR1 cells. d) Schematic of how the different parts of the lymph node were defined for e), which shows the changes in localization of XCR1 cells from 0 to 24 hours. f) Schematic of the metric used to assess dendritic cell clustering. g) Histograms of DC cluster density at locations of different types of T cells. h) Mean fraction of detected XCR1 cells within distance of different types of T cells. Shaded area represents standard error. i) Mean percent of XCR1 cells within 100 μm vs. distance to cortex at 0 and 24 hours. Error bars represent bootstrapped 95% confidence interval. j) Histogram of XCR1 cell distances to cortex at 0 and 24 hours. k) Percent of XCR1 cells within 100 μm vs. distance to HEVs at 0 and 24 hours. Error bars represent bootstrapped 95% confidence interval. l) Histogram of XCR1 cell distances to HEVs at 0 and 24 hours.

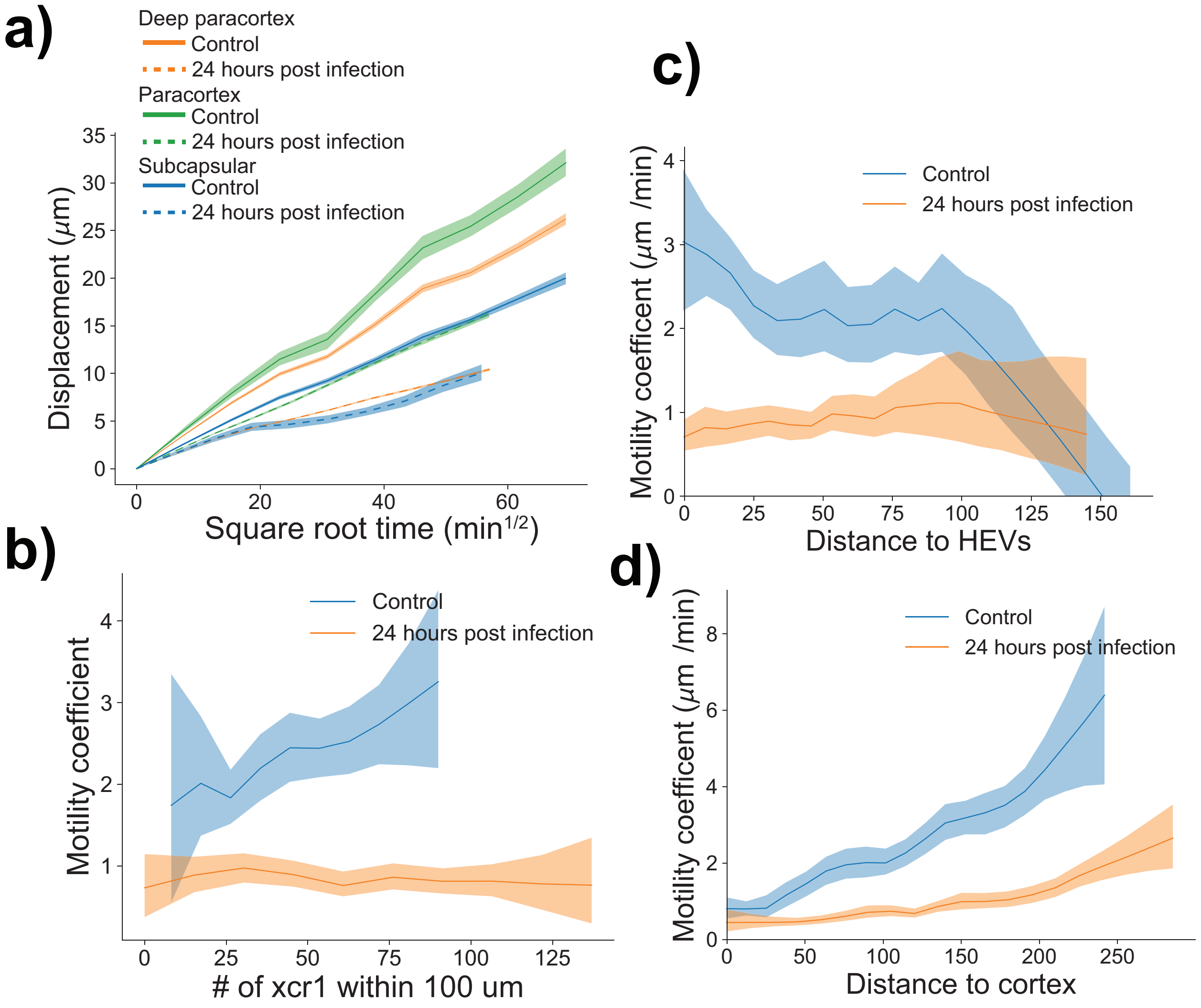

Supplementary Figure 11: Dendritic cell motility changes in different anatomical locations a) Mean displacement vs. square root time plots for dendritic cells in different parts of the lymph node at 0 and 24 hours. b) Mean dendritic cell motility coefficients vs the number of other dendritic cells within 100 μm. c) Mean motility coefficient vs. distance to high endothelial venules. d) Mean motility coefficient vs. distance to cortex. Shaded regions in all plots represent bootstrapped 95% confidence intervals.
