# Supplementary material for: Learned adaptive multiphoton illumination microscopy for large-scale immune response imaging
Source: Nat Commun. 2021 Mar 26;12:1916. doi: 10.1038/s41467-021-22246-5 (PMC7997974; doi:10.1038/s41467-021-22246-5)
Supplement: Supplementary file 13 — Reporting Summary [file 41467_2021_22246_MOESM13_ESM.pdf]

nature research

Corresponding author(s): Henry Pinkard
Last updated by author(s): 2020-8-24

Reporting Summary

Nature Research wishes to improve the reproducibility of the work that we publish. This form provides structure for consistency and transparency in reporting. For further information on Nature Research policies, see our Editorial Policies and the Editorial Policy Checklist.

Statistics

For all statistical analyses, confirm that the following items are present in the figure legend, table legend, main text, or Methods section.

n/a	Confirmed	
[ ]	[x]	The exact sample size (n) for each experimental group/condition, given as a discrete number and unit of measurement
[ ]	[x]	A statement on whether measurements were taken from distinct samples or whether the same sample was measured repeatedly
[x]	[ ]	The statistical test(s) used AND whether they are one- or two-sided<br>*Only common tests should be described solely by name; describe more complex techniques in the Methods section.*
[x]	[ ]	A description of all covariates tested
[x]	[ ]	A description of any assumptions or corrections, such as tests of normality and adjustment for multiple comparisons
[ ]	[x]	A full description of the statistical parameters including central tendency (e.g. means) or other basic estimates (e.g. regression coefficient) AND variation (e.g. standard deviation) or associated estimates of uncertainty (e.g. confidence intervals)
[x]	[ ]	For null hypothesis testing, the test statistic (e.g. F, t, r) with confidence intervals, effect sizes, degrees of freedom and P value noted<br>*Give P values as exact values whenever suitable.*
[x]	[ ]	For Bayesian analysis, information on the choice of priors and Markov chain Monte Carlo settings
[x]	[ ]	For hierarchical and complex designs, identification of the appropriate level for tests and full reporting of outcomes
[x]	[ ]	Estimates of effect sizes (e.g. Cohen's d, Pearson's r), indicating how they were calculated

Our web collection on statistics for biologists contains articles on many of the points above.

Software and code

Policy information about availability of computer code

Data collection	Data was collected with the Micro-Magellan plugin to Micro-Manager 1.4. Specifically, a version of Micro-Magellan which had modifications for the two photon system we were using, and the ability to use neural net modulated excitation. The source code for this version is available here (https://github.com/henrypinkard/Pygellan/tree/twophoton). A custom device adapter was compiled in order to control the spatial light modulator--source code found here (https://github.com/micro-manager/micro-manager/tree/master/DeviceAdapters/TeesnySLM).<br><br>Since performing the original experiment, an updated, streamlined codebase for data collection was created, which can be found here: https://doi.org/10.5281/zenodo.4314107<br><br>Imaris 7.6.5 was used for data visualization. Matlab r2018a through the Imaris XT bridge was used for the cell tracking and active learning described in the manuscript. SciPy 1.2 was used for analysis and production of figures
Data analysis	All other code including data analysis code can be found on Zenodo https://doi.org/10.5281/zenodo.4315851

For manuscripts utilizing custom algorithms or software that are central to the research but not yet described in published literature, software must be made available to editors and reviewers. We strongly encourage code deposition in a community repository (e.g. GitHub). See the Nature Research guidelines for submitting code & software for further information.

Data

Policy information about availability of data

All manuscripts must include a data availability statement. This statement should provide the following information, where applicable:

- Accession codes, unique identifiers, or web links for publicly available datasets
- A list of figures that have associated raw data
- A description of any restrictions on data availability

The imaging data that supports this findings of this study is available in figshare with identifier https://doi.org/10.6084/m9.figshare.12841781.v1

Field-specific reporting

Please select the one below that is the best fit for your research. If you are not sure, read the appropriate sections before making your selection.

[x] Life sciences	[ ] Behavioural & social sciences	[ ] Ecological, evolutionary & environmental sciences

For a reference copy of the document with all sections, see nature.com/documents/nr-reporting-summary-flat.pdf

Life sciences study design

All studies must disclose on these points even when the disclosure is negative.

Sample size	Three replicates (i.e. different animals) were obtained for each intravital imaging condition, across two-three animals. The same behavior was observed across all replicates. within each sample >= 10 (and usually closer to 1000) cells were observed and tracked, as detailed in the supplementary information. The bootstrapped error bars used in this study generally show minimal reduction in standard error with sample sizes greater than this. Since no hypothesis tests were performed in this study, there are no a priori sample size thresholds to be met. Three replicates were demonstrated consistent behavior across animals, and no claims are made in the paper that are not commensurate with number of replicates performed.
Data exclusions	No data were excluded. The one 3D video in each set of replicates (i.e. different animal) that was chosen for quantification was picked on the basis of the length of the video (i.e. the longest) and the quality of the data produced (i.e. cells visible with the highest signal to noise ratio).
Replication	Three replicates (i.e. different animals) were obtained for each intravital imaging condition, across two-three animals. Any conclusions highlighted in the text (i.e. the aggregate migration behavior of cells) was consistent across all observed replicates
Randomization	Littermate mice were used as control and treatment groups. Internal control groups were used whenever possible (i.e. polyclonal cells). Animals were allocated randomly into control and treatment groups
Blinding	Blinding was not relevant to this study due to the presence of internal controls (i.e. polyclonal cells) and automated analysis methods that were applied with the same parameters across control and treatment conditions. Furthermore, the biological conclusions presented were exploratory in nature, and were not designed to test a specific hypothesis for which prior beliefs were held

Reporting for specific materials, systems and methods

We require information from authors about some types of materials, experimental systems and methods used in many studies. Here, indicate whether each material, system or method listed is relevant to your study. If you are not sure if a list item applies to your research, read the appropriate section before selecting a response.

Materials & experimental systems

n/a	Involved in the study
[ ]	[x] Antibodies
[x]	[ ] Eukaryotic cell lines
[x]	[ ] Palaeontology and archaeology
[ ]	[x] Animals and other organisms
[x]	[ ] Human research participants
[x]	[ ] Clinical data
[x]	[ ] Dual use research of concern

Methods

n/a	Involved in the study
[x]	[ ] ChIP-seq
[x]	[ ] Flow cytometry
[x]	[ ] MRI-based neuroimaging

Antibodies

Antibodies used	1) Peripheral Node Addressin Antibody (MECA-79) [Alexa Fluor® 647]: Novus Biologicals; catalog no. NB100-77673AF647<br>2) Peripheral Node Addressin Antibody (MECA-79) [Alexa Fluor® 488]: Novus Biologicals; catalog no. NB100-77673AF488
Validation	No validation has been done as only secondary antibodies have been used in the study.

Animals and other organisms

Policy information about studies involving animals; ARRIVE guidelines recommended for reporting animal research

Laboratory animals	1) Mus musculus; C57BL/6 (stock no:000664; Jackson Laboratory); male or female; 6-12 weeks<br>2) Mus musculus; TCRa knock-out, LCMV P14 specific transgenic mice (MMRRC stock no:37394-JAX; Jackson Laboratory); male or female; 6-12 weeks<br>3) Mus musculus; OT-1 (stock no:003831; Jackson Laboratory); male or female; 6-12 weeks<br>4) Mus musculus; OT-2 (stock no:004194; Jackson Laboratory); male or female; 6-12 weeks<br>5) Mus musculus; ubiquitin-GFP (stock no:004353; Jackson Laboratory); male or female; 6-12 weeks<br>6) Mus musculus; C57BL/6 background CD2 RFP; male or female; 6-12 weeks<br>7) Mus musculus; C57BL/6 background XCR1 Venus; male or female; 6-12 weeks<br><br>Mice were maintained at a constant humidity between 30-70% and temperature 68-79 degrees Fahrenheit, under a 12-h light/ dark cycle and had free access to food and water until experiment initiation.
Wild animals	No wild animals were used in the study,
Field-collected samples	No field collected samples were used in the study.
Ethics oversight	All mice were treated in accordance with the regulatory standards of the National Institutes of Health and American Association of Laboratory Animal Care and were approved by the UCSF Institution of Animal Care and Use Committee (IACUC approval: AN170208). All mice were purchased for acute use or maintained under specific pathogen-free conditions at the University of California, San Francisco Animal Barrier Facility.

Note that full information on the approval of the study protocol must also be provided in the manuscript.
