# Supplementary material for: Learned adaptive multiphoton illumination microscopy for large-scale immune response imaging
Source: Nat Commun. 2021 Mar 26;12:1916. doi: 10.1038/s41467-021-22246-5 (PMC7997974; doi:10.1038/s41467-021-22246-5)
Supplement: Supplementary file 14 — Description of Additional Supplementary Files [file 41467_2021_22246_MOESM14_ESM.pdf]

Title: Supplementary movie 1:
Description: Visible photodamage caused by over illumination. A lymph node explant imaged with an inappropriate adaptive illumination scheme. The parts of the cortex on the bottom of the frame receive too much excitation power, resulting in the parenchyma bursting out from the lymph node cortex.

Title: Supplementary movie 2:
Description: Standard candle training data. Inguinal lymph node seeded with GFP lymphocytes imaged with randomized excitation power to generate standard candle training data

Title: Supplementary movie 3:
Description: ex vivo lymph node imaged with adaptive excitation. Inflamed mediastinal lymph node with adoptively transferred lymphocytes labelled with GFP, RFP, eFluor670 imaged using adaptive excitation.

Title: Supplementary movie 4:
Description: LAMI applied to an irregularly shaped lymph node. A multi-lobed, aspherical lymph node imaged by LAMI. Clear contrast visible throughout the volume, with no evidence of over- excitation or photo-damage. Lymph node contains endogenous XCR1-Venus cells (yellow) and adoptively transferred lymphocytes with GFP (green) and eFluor670 (pink).

Title: Supplementary movie 5:
Description: Correction of transverse movements within Z-stack. single Z-stack with transverse movement artifact caused by breathing (left) and with optimized corrections (right)

Title: Supplementary movie 6:
Description: 3D volume timelapse before and after motion correction and registration. Timelapse before and after registration, motion corrections and stitching

Title: Supplementary movie 7:
Description: Cleared lymph node (no immunization). Cleared lymph node showing positioning of XCR1+ cells (yellow) when in uninfected condition

Title: Supplementary movie 8:
Description: Cleared lymph node 24 hours after immunization. Cleared lymph node showing positioning of XCR1+ cells (yellow) 24 hours after immunization with LPS

Title: Supplementary movie 9:
Description: In vivo immune response 24 hours after immunization.

Title: Supplementary movie 10:
Description: In vivo whole lymph node dynamics 5 hours after immunization

Title: Supplementary movie 11:
Description: In vivo cell dynamics 5 hours after immunization and tracked dendritic cells showing clustering behaviour
